# Food Insecurity Among College Students with and without Medical Disorders at a University in Appalachia

**DOI:** 10.13023/jah.0202.04

**Published:** 2020-04-15

**Authors:** Laura H. McArthur, Melissa Gutschall, Kimberly S. Fasczewski, Anna Jackson

**Affiliations:** Department of Nutrition and Health Care Management, Appalachian State University; Department of Nutrition and Health Care Management, Appalachian State University; Department of Health and Exercise Science, Appalachian State University; Department of Nutrition and Health Care Management, Appalachian State University

**Keywords:** Appalachia, college students, food insecurity, money-spending behaviors, food access barriers

## Abstract

**Objective:**

This study compared severity of food insecurity, characteristics, and behaviors of college students with and without diagnosed medical disorders.

**Design:**

Data were collected using a cross-sectional online questionnaire. Variables measured were food security status, disorders, coping strategies, and perceived barriers to food access. Descriptive and inferential statistics examined associations and compared groups. Statistical significance was p≤0.05.

**Setting:**

Data were collected at Appalachian State University in North Carolina.

**Participants:**

The sample was 247 food insecure students, of whom 60% were females, 50% 3rd- and 4th-year students, and 75% whites.

**Results:**

Medical disorders were reported by 67.2% of food insecure students, and a greater proportion of students with than without disorders experienced very low food security (63.3% vs. 43.2%, p=0.003). The most common disorder categories were psychiatric (40.5%) and gastrointestinal (31.6%). Characteristics of food insecure students with disorders included female gender, suboptimal academic performance, employed, off-campus residence. Coping strategies used by students with and without disorders, respectively, to improve food access, included brought food back to school after visiting family, friends, significant others (90.9% vs. 63.0%) and ate less healthy food so you could eat more (77.7% vs. 49.4%). Perceived barriers among students with disorders included feel overwhelmed making food choices (12.7%) and meal plan runs out (10.2%). Food insecure students with disorders made greater use of coping strategies and identified more perceived barriers.

**Conclusions:**

Food insecure students with disorders experienced more severe food deprivation and require multidimensional food assistance programs beyond those generally available on college campuses.

## INTRODUCTION

Food security is defined as having regular access, in socially acceptable ways, to nutritionally adequate and safe food that supports an active and healthy life.^1^ The Adult Food Security Survey (AFSS) is administered annually to determine the food security status of the adult population.^1^ Respondents are classified along a continuum from high to very low food secure depending on the number of affirmative answers to ten questions that assess the quantity, quality, variety, and desirability of their food supply.^2^ The four levels of food security are: high (no food-access problems), marginal (worry or anxiety about food sufficiency, with little or no changes in diet quality or eating pattern), low (reduced diet quality, variety, or desirability, with little or no change in the eating pattern), and very low (decreased quantity and quality of the food supply and reduced food intake). Persons whose scores fall in the high or marginal categories are classified as food secure and those whose scores fall in the low or very low categories are classified as food insecure.^1^

Prevalence data from the USDAERS indicate that approximately 37.2 million adults were food insecure at some time during 2018, including 9.5 million who were very low food secure.^2^ Households in 2018 with food insecurity rates above the national average of 11.1% were those with children, single parent households, men and women living alone, black, non-Hispanics, Hispanics, residents of the southern and southeastern regions, and households with incomes below 185% of the poverty threshold.^2^ The unfavorable health outcomes associated with prolonged food insecurity include obesity and related chronic diseases,^3^ mental health disorders,^4^ impaired cognitive functionality,^5^ and poor growth and development in children and adolescents.^6^

Evidence from 2- and 4-year public and private post-secondary institutions nationwide indicates that food insecurity is a public health problem among U.S. college students.^7^ Research conducted at seven colleges and universities in the Appalachian region found rates of student food insecurity ranging from 22.4% to 51.8%.^8^ The present study was conducted at Appalachian State University (AppState) located in the western region of North Carolina where food insecurity rates at the county level range from 13% to 16.8%.^9^ During the spring 2016 semester the rate of student food insecurity at AppState was 46.2%.^10^ The sociodemographic characteristics most frequently associated with food insecure college students include: older age, receiving food assistance, having less money to buy food, identifying with a minority race/ethnic group, being employed while in school, on-campus residence, having lower self-efficacy for cooking cost-effective, nutritious meals, having less time to prepare food, and suboptimal academic performance as reflected in a low grade point average (GPA).^11^–^13^ Additionally, food insecure college students show higher rates of depression, anxiety, and stress compared to their food secure peers.^14^,^15^

The research concerning college student food insecurity has focused primarily on measuring prevalence rates and identifying sociodemographic and behavioral characteristics.^10^–^13^ To our knowledge, few studies have measured rates of food insecurity among college students who are challenged with physical, mental health, or learning disorders. The sparse research on this topic has thus far examined associations between food insecurity and depression, anxiety, and stress.^13^–^15^ These investigators have recommended that campus administrators implement multifaceted interventions that improve access to healthy foods and offer physical and mental health and tutoring services to promote greater likelihood of student success. The present study was conducted to contribute to our understanding of the college student food insecurity problem by posing the following questions: (1) Do food insecure students with medical or learning disorders experience more severe food insecurity than their food insecure peers without disorders? and (2) Do food insecure students with disorders differ from their food insecure peers without disorders in their academic performance or in their food access behaviors?

The hypotheses tested were: (1) A significantly greater proportion of food insecure students with disorders will report experiencing very low food security than their food insecure peers without disorders, (2) there will be a significant negative correlation for the food insecure students with disorders between their food security scores and their GPA, and (3) There will be significant positive correlations for the food insecure students with disorders between their food security scores and their scores on the perceived barriers to food access and coping behaviors scales. The rationale underlying this research was to determine whether there exists a cohort of food insecure students who might, due to their medical and learning disorders, need different types of assistance accessing food beyond the usual campus services.^16^ Since learning disorders are often rooted in neurologic conditions,^17^ the six learning disorders were included in the neurological category of medical disorders. Therefore, throughout this manuscript, the term disorders will refer to physical and mental health and learning disorders.

## METHODS

### Participants and Recruitment

A nonprobability, random, computer-generated sample of 6000 students was sent electronic recruitment letters during the spring, 2018 semester. Inclusion criteria were any gender identity, undergraduate status, at least 18 years of age, on- or off-campus residence, and any race or ethnicity. Recruitment letters were sent as two email blasts with reminders at one and two weeks (3000 emails/blast).^18^ Students who wished to participate clicked a link in the recruitment letter that took them to a screen showing the elements of informed consent and the initial questionnaire items. Students who completed the questionnaire could click a link to a detached file to enter a drawing for a $50 Amazon.com gift card. This study was deemed exempt by the Institutional Review Board at the university.

### Survey Instrument

Data were collected using an anonymous online questionnaire administered through Qualtrics survey software (Qualtrics, Provo UT, 2018). The questionnaire included 46 close-ended items and took participants 20–25 minutes to complete. The students’ food security status was determined by calculating their scores on the 10-item USDAERS Adult Food Security Survey (AFSS).^1^ The stem for these questions was: “As a student at App State” to reflect usual food access since enrolling at the university. Information concerning diagnosed disorders was collected from a list of 74 conditions identified in a National College Health Assessment report published by the American College Health Association.^19^ These conditions were assigned to categories based on the classification of Escott-Stump^20^ as follows: 17 psychiatric, 12 gastrointestinal, 9 neurologic, 7 musculoskeletal, 6 learning, 5 immunologic, 3 cardiovascular, 3 endocrine, 2 autism spectrum, 2 pulmonary, 2 weight-related, 1 hematologic, and 5 “other.” Students checked those disorders for which they had been diagnosed by a medical professional. Students also self-rated their health by checking poor, fair, good, or excellent.

Food access was assessed with a perceived-barriers and a coping-behaviors scale. Perceived barriers to food access on and off-campus were identified from a checklist of 26 possible barriers grouped as follows: 7 food access, 6 practical concerns, 5 knowledge, 5 personal concerns, and 3 affective. The coping scale consisted of 27 food access behaviors, and the students checked never, seldom, sometimes, or often to estimate how frequently they used each during a typical semester. The Cronbach alpha reliability coefficient for this scale was 0.81. The perceived barriers scale was developed by the authors and the coping behaviors scale was developed with guidance from pertinent literature.^10^–^14^

The questionnaire concluded by eliciting information about sociodemographic, anthropometric, academic, and cooking variables. The academic variables included grade point average (GPA) and a 4-item academic progress scale where the students self-rated their performance on (1) overall progress in school, (2) class attendance, (3) attention span in class, and (4) understanding of concepts taught, by checking poor, fair, good, or excellent.

Content validity was determined by two nutrition professors and the questionnaire was pilot tested online with 42 students enrolled in an introductory nutrition class that meets a general education requirement. Student feedback indicated that an appropriate number of questions was displayed per screen and that the buttons worked well. The only change was the addition of polycystic ovarian syndrome to the list of medical disorders.

### Statistical Analysis

Data were analyzed using the Statistical Package for the Social Sciences (SPSS version 25.0, IBM Corp., Armonk NY, 2017). Frequency counts and percentages were calculated on all variables. Food security status was determined using the USDAERS scoring system for the AFSS such that zero affirmative responses reflected high, 1–2 marginal, 3–5 low, and 6–10 very low food security; students in the high and marginal groups were classified as food secure while those in the low and very low groups were classified as food insecure.^1^ Only the responses from food insecure students were analyzed to address the study questions and hypotheses.

Data from the perceived barriers checklist were scored by summing the number of times each barrier was selected and rank-ordering the barrier categories and the barriers within each category. The coping behaviors scale was scored by assigning 1 point to never, 2 to seldom, 3 to sometimes, and 4 to often responses, with possible scores ranging from 27 to 108 points and from 15 to 60 points, respectively. The academic progress scale was scored by allotting 1 point to poor, 2 to fair, 3 to good, and 4 to excellent responses, with possible scores ranging from 4 to 16 points. The items that assessed perceived health status and cooking skills were similarly scored, with possible scores ranging from 1 to 4 points.

Correlational analyses assessed the strength of associations between food security scores and GPA, and between food security scores and scores on the academic progress, perceived barriers, and coping behaviors scales. Chi-square analyses compared proportions of food insecure students with and without disorders regarding sociodemographics, perceived health, frequency of cooking, and self-rated cooking skills. Independent-samples t-tests compared the means of the two groups on BMI, GPA, and scores on the academic progress and coping behaviors scales. Statistical significance was p≤0.05.

## RESULTS

### Profile of Food Insecure Students

Questionnaires were submitted by 493 of the 6000 recruited students (8.2%), of whom 247 (50.1%) were food insecure. Comparisons indicated that the sample closely resembled the distributions of gender, race, ethnicity and academic year for the student enrollment at App State during the period of data collection. Table 1 (see Additional Files) shows frequencies and percentages for the entire sample of food insecure students based on sociodemographic, health, and cooking variables.

In summary, ~60% were females with a mean age of 21.4 years (± 2.74, range 18 to 40); ~75% self-classified as non-Hispanic white; ~70% were undergraduates; and ~60% lived on-campus. Additionally, ~50% of the students held one or more part-time jobs, ~60% were financial aid recipients, ~60% had a monthly income of less than $500, and ~60% did not participate in a university meal plan. The students’ mean BMI was 25.09 kg/m^2^ (±5.9, range 15.78 to 48.08). This mean BMI classifies students in the overweight category, while the range indicates that students’ weight varied from underweight to extremely obese. Yet, ~50% of students perceived their health as either good or excellent. Last, ~75% sometimes or often cooked for themselves or others and ~50% perceived their cooking skills as good or excellent.

### Diagnosed Disorders of Food Insecure Students

Diagnosed medical disorders were reported by 166 (67.2%) of the 247 food insecure students, of whom 160 (96.4%) reported one or more physical or mental health disorders and six (3.6%) reported one or more learning disorders. The disorders reported most often were depression (61, 36.7%); generalized anxiety disorder (46, 27.7%); acid reflux (39, 23.5%); back pain (34, 20.5%); migraine headache (22, 13.3%); and attention deficit hyperactivity disorder (22, 13.3%).

### Comparisons of Food Insecure Students with and Without Disorders

Table 1 (see Additional Files) also shows the frequencies, percentages, and chi-square comparisons for the two groups of food insecure students based on sociodemographic, health, and cooking variables. There were significantly greater proportions of food insecure males and females with than without disorders (p=0.028). Significantly greater proportions of students with disorders were low and very low food secure compared to their peers without disorders (p=0.003). A greater proportion of food insecure students with disorders cooked for themselves or others sometimes or often, and no significant difference was found between the proportions who rated their cooking skills as excellent or good. No significant differences were found between the mean BMIs of the two groups or between the proportions who rated their perceived health as excellent/good or fair/poor.

No significant differences were found between the mean GPAs of students with and without disorders, respectively (3.34 ±0.50 vs. 3.36 ±0.49) or between their mean scores on the academic progress scale (12.54 ± 2.40 vs. 13.02 ± 2.66) out of a possible 16 points. However, the students with disorders scored significantly lower (p=0.021) on the scale item concerning attention span in class. A significant negative correlation, although modest, was found only for the students with disorders between their food security scores and their GPAs (r = −0.201, p=0.017) and between their food security scores and their scores on the academic progress scale (r = −0.195, p=0.016).

Table 2 (see Additional Files) shows frequency counts and percentages for barriers to accessing food at on and off-campus locations identified by the food insecure students with and without disorders.

**The barriers for all students at both locations centered around, time, cost, preparation, and food preferences.** The on-campus barriers selected most often by students with disorders were: meal plan runs out, foods are not always healthy or nutritious, and available foods do not taste good; those selected most often by students without disorders were: meal plan runs out, food preparation is inconvenient, and foods are not always healthy or nutritious. The off-campus barriers selected most often by students with disorders were: feel overwhelmed and stressed planning meals or making food choices, food preparation is inconvenient, and don’t have time to purchase food; those identified most often by the students without disorders were: don’t know how to ask for help, feel overwhelmed and stressed planning meals or making food choices, and food preparation is inconvenient. Among students with disorders specifically, significant positive correlations were found between their food security scores and the number of perceived barriers to food access for both on and off-campus locations (r =0.260 and p=0.050 for both locations).

Table 3 (see Additional Files) shows the frequency counts and percentages of the coping behaviors used by the food insecure students with and without disorders.

The behavior receiving the greatest number of sometimes or often responses from both groups was brought food back to school after visiting family, friends, or significant others. The other behaviors identified most often by the students with disorders were ate less healthy foods to eat more and ate smaller portions, while those selected by the students without disorders were ate smaller portions and planned menus. No significant differences were found between the mean coping scale scores for the students with and without disorders, respectively (56.02, ±11.62, range 14 to 97 points vs. 53.31 ±12.08, range 34 to 82 points) out of a possible 108 points. Significant positive correlations were found for the students with and without disorders, respectively, between their academic progress and coping behaviors scale scores (r =0.449, p<0.01 vs. r = 0.582, p<0.01).

## IMPLICATIONS

As hypothesized, a significantly greater proportion of food insecure students with disorders experienced very low food security compared to their peers without disorders. The most frequently identified disorders were from the psychiatric category, as reported by other investigators,^13^–^15^ but our participants also reported diagnoses for gastrointestinal and neurological conditions not previously identified for food insecure college students. These findings suggest a need for a multifaceted approach to decreasing the occurrence of food insecurity in this population. Accordingly, campus administrators are encouraged to continue supporting food pantries as a temporary measure for facilitating food access, but should also implement permanent policies and programs that make nutritious foods more affordable for all food insecure students, and augment existing services that offer counseling, referrals, and assistance with management of medical conditions for the cohort with disorders.

The food insecure students with and without disorders felt overwhelmed and stressed when planning meals and making food choices. Despite their challenges, these students need to consume an adequate daily diet to maintain their nutrient reserves to promote optimum physical and cognitive functionality.^3^,^7^ Access to an adequate diet is even more urgent for the students with disorders who are more severely affected by food insecurity. However, the types of food assistance currently available at the university may not adequately serve these special needs students. Therefore, student leaders, health educators, academic advisers, and campus administrators are encouraged to reach out to these affected students through offices of disability support services and student life to identify the forms of assistance that these students would find most helpful. Once these complementary services are identified, partnerships could be established with sororities and fraternities, student dietetic associations, and public health, health promotion, social work, and nursing clubs to plan and implement these activities.

Volunteers from these organizations could also serve their peers by joining them for meals, grocery shopping, and meal preparation as a source of social support. Administrators could help by initiating policy changes that facilitate the establishment of campus community gardens, negotiate for less expensive campus meal plans, implement programs that make unused meal plan funds available to students whose plans have run out, offer a wider variety of affordable, nutritious food options at on-campus locations, and support greater opportunities for on-campus student employment.^21^ Such policies and programs would allow food insecure students to spend less money on food and better afford the myriad of school and living expenses accompanying the transition from home to campus life. These expenses, along with the costs of diagnostic tests and treatment modalities may have contributed to more severe food insecurity among the students with disorders.

Another finding for consideration by campus administrators was that measures of academic performance declined as the students’ food insecurity became more severe. This relationship was more pronounced, although modestly, among the students with disorders, as hypothesized. Additionally, this cohort perceived more barriers to food access on and off campus as their food security status worsened. These findings offer opportunities for introducing food security education to help students overcome these barriers and reduce their risk for food insecurity. Such activities could teach budgeting, menu planning, food purchasing and preparation, use of leftover food, gardening, and advocacy.

No significant difference was found between the mean coping behaviors scale scores of the students with and without disorders, but significant positive correlations were found between their food security scores and coping behaviors scale scores. This implies that as the students’ food security status deteriorated they used a greater number of these coping behaviors and used them more often. Given that this trend applied to the students with and without disorders, a circumstance other than the presence of a disorder, such as financial constraints, could have prompted adoption of these behaviors. The two most frequently used coping behaviors were bringing food back to school after visiting family, friends, or significant others and eating smaller portions. This implies that the students avoided hunger by relying on their social support systems and by rationing food into smaller portions to stretch their food supply. A third frequent behavior was eating less healthy foods to eat more, suggesting regular consumption of an energy-dense diet featuring refined grains and added sugars and fats. Adoption of such a diet over the span of a college career is conducive to becoming overweight, developing chronic conditions associated with excess adiposity, and compromising nutrient reserves.^3^

Several limitations prevent the generalizability of the present findings to all food insecure college students in Appalachia, including a modest sample size, overrepresentation of females and whites, data collection on a single campus, and self-reporting of all measures. The number of students reporting learning disorders should be interpreted with caution since this may have been underreported, perhaps because they resided in an environment where learning is expected. Despite these limitations, the findings contribute to the literature by identifying a cohort of food insecure students with diagnosed disorders whose health and academic success could be in jeopardy unless novel types of assistance are made available. In this regard, future research with food insecure students challenged with medical disorders is needed to identify specific food assistance and health services that they believe would improve their likelihood of academic success and retention.

**SUMMARY BOX**

**What is already known on this topic?** Research indicates that food insecurity is a common problem among college students in Appalachia, and several studies have identified significant positive correlations between food insecurity and scores on depression, anxiety, and stress scales.

**What is added by this report?** This research found that the cohort of college students with diagnosed medical disorders experienced more severe food insecurity compared to their food insecure peers without such disorders. Moreover, the academic performance of this group, as reflected in their grade point average and their score on the academic progress scale was lower, although not significantly, compared to the students without disorders. Lastly, the students with disorders identified more perceived barriers to food access and used coping behaviors more frequently than those without disorders.

**What are the implications for future research?** Research with food insecure students challenged with medical disorders is needed to identify specific food assistance and health services that they believe would improve the likelihood of their academic success and retention.

## Supplementary Information



## References

[1] United States Department of Agriculture Economic Research Service Definitions of Food Security https://www.ers.usda.gov/topics/food.../food-security.../definitions-of-food-security.aspx

[2] United States Department of Agriculture Economic Research Service Key Statistics and Graphics https://www.ers.usda.gov/.../food.../food-security-in-the-us/key-statistics-graphics.aspx

[3] Parker ED Widome R Nettleton JA Pereira MA Food security and metabolic syndrome in U.S. adults and adolescents: Findings from the National Health and Nutrition Examination Survey, 1999–2006 Ann Epidemiol 2010 20 364 70 10.1016/j.annepidem.2010.02.009 20382337 PMC5842796

[4] Burke MP Martini LH Çayır E Severity of Household Food Insecurity Is Positively Associated with Mental Disorders among Children and Adolescents in the United States J Nutr 2016 146 2019 26 10.3945/jn.116.232298 27581581

[5] Wong JC Scott T Wilde P Li YG Tucker KL Gao X Food Insecurity is associated with Subsequent Cognitive Decline in the Boston Puerto Rican Health Study J Nutr 2016 145 1740 45 10.3945/jn.115.228700 PMC4997276 27466603

[6] Gross RS Mendelsohn AL Food insecurity during early childhood: Marker for disparities in healthy growth and development Pediatr 2019 144 4 pii:e20192430 10.1542/peds.2019-2430 Epub 2019 Sep 9 31501234

[7] Freudenberg N Goldrick-Rad S Poppendieck J College students and SNAP: The new face of food insecurity in the United States Am J Public Health 2019 109 12 1652 58 10.2105/AJPH.2019.305332. 31622149 PMC6836795

[8] Hagedorn RL McArthur LH Hood LB Expenditure, Coping, and Academic Behaviors among Food-Insecure College Students at 10 Higher Education Institutes in the Appalachian and Southeastern Regions Curr Dev Nutr 2019 3 nzz058 10.1093/cdn/nzz058 31149651 PMC6536735

[9] Map the Meal Gap Feeding America https://map.feedingamerica.orgcounty/2017/overall/north-carolina/

[10] McArthur LH Ball L Danek AC Holbert D A High Prevalence of Food Insecurity Among University Students in Appalachia Reflects a Need for Educational Interventions and Policy Advocacy J Nutr Educ Behav 2018 50 6 564 72 10.1016/j.jneb.2017.10.011 29242138

[11] Bruening M Argo K Payne-Sturges D Laska MN The struggle is real: A systematic review of food insecurity on postsecondary campuses J Acad Nutr Diet 2017 117 1767 91 10.1016/j.jand.2017.05.002 28754200 PMC6901286

[12] Gaines A Robb CA Knol LL Examining the role of financial factors, resources and skills in predicting food security status among college students: Food security and resource adequacy Int J Consum Stud 2014 38 374 84 10.1111/ijcs.12110

[13] Raskind IG Haardörfer r Berg CJ Food insecurity, psychosocial health and academic performance among college and university students in Georgia, USA Public Health Nutr 2019 22 476 85 10.1017/S1368980018003439 30724722 PMC6366643

[14] Meza A Altman E Martinez S Leung CW “It’s a feeling that one is not worth food”: A qualitative study exploring the psychosocial experience and academic consequences of food insecurity among college students J Acad Nutr Diet 2019 119 10 1713 21 10.1016/j.jand.2018.09.006 30553586 PMC6561835

[15] Wall-Bassett E Li Y Matthews F The Association of Food Insecurity and Stress Among College Students in Rural North Carolina J Nutr Educ Behav 2017 49 S75 10.1016/j.jneb.2017.05.218

[16] Davis H Sisson SB Clifton S A call for evidence to support food security interventions on college campuses J am Coll Health 2020 Jan 23 1 3 10.1080/07448481.2019.1705829 31971893

[17] Learning Disabilities Association of America What are Learning Disabilities? https://www.ldaamerica.org/types-of-learning-disabilities/

[18] Dillman DA Smyth JD Christian LM Internet, Phone, Mail, and Mixed-Mode Surveys: The Tailored Design Method 4th ed Hoboken John Wiley and Sons, Inc 2014

[19] American College Health Association American College Health Association-National College Health Assessment: Reference Group Data Report Baltimore, MD American College Health Association 2008 10.3200/acha/57.5.477-488

[20] Escott-Stump S Nutrition and Diagnosis-Related Care 8th ed Philadelphia Lippincott Williams & Wilkins 2015

[21] Fetter A Gilboy M Addressing food insecurity on college campuses: The food pantry and beyond J Acad Nutr Diet 2018 118 A160 10.1016/j.jand.2018.08.126

