# Supplementary material for: Food Insecurity Among College Students with and without Medical Disorders at a University in Appalachia
Source: J Appalach Health. 2020 Apr 15;2(2):36–48. doi: 10.13023/jah.0202.04 (PMC9138724; doi:10.13023/jah.0202.04)
Supplement: Supplementary file 1 [file 1046Tables2.2.4-MacArthur.pdf]

Table 1. Sociodemographic, Health and Cooking Characteristics of Food Insecure Students with (n=166) and without (n=81) Disorders and Entire Food Insecure Sample (n=247)

	Entire Food Insecure Sample		Food Insecure Students with Disorders		Food Insecure Students without Disorders		p-value Disorder vs No Disorder
**Characteristic**	**n**	**%**	**n**	**%**	**n**	**%**	
**Food security classification**							0.003*
Low Food Secure	107	43.3	61	36.7	46	56.8	
Very Low Food Secure	140	56.7	105	63.3	35	43.2	
**Gender**							0.028*
Male	55	22.3	33	19.9	22	27.2	
Female	150	60.7	115	69.3	35	43.2	
Other	4	1.6	4	2.4	0	0	
Missing	38	15.4	14	8.4	24	29.6	
**Race/ethnicity**							0.263
African American, not of Hispanic origin	9	3.6	7	4.2	2	2.5	
African American, White	3	1.2	2	1.2	1	1.2	
American Indian	4	1.6	2	1.2	2	2.5	
Asian	1	0.4	0	0	1	1.2	
Hispanic	12	4.9	10	6.0	2	2.5	
White, not of Hispanic origin	179	72.5	128	77.1	51	63.0	
Other	3	1.2	2	1.2	0	0	
**Academic year**							0.109
1/2	60	24.3	42	25.3	18	22.2	
3/4	117	47.4	84	50.6	33	40.7	
Graduate student	25	10.1	19	11.4	6	7.4	
Other	2	0.8	2	1.2	0	0	
Missing	43	17.4	19	11.4	24	29.6	
**Residency**							0.650
On-campus	49	19.8	34	20.5	15	18.5	
Off-campus	154	62.3	112	67.5	42	51.9	
Missing	44	17.8	20	12.0	24	29.6	
**Employment status**							0.541
Unemployed	58	23.5	38	26.0	20	35.1	
One-or-more part-time jobs	130	52.6	97	66.4	33	57.9	
One full-time job	6	2.4	5	3.4	1	1.8	
Other	9	3.6	6	4.1	3	5.3	
Missing	44	17.8	20	12.0	24	29.6	

**Financial aid recipient**							0.341
Yes	156	63.2	115	69.3	41	50.6	
No	48	19.4	32	19.3	16	19.8	
Missing	43	17.4	19	11.4	24	29.6	
**Personal monthly income**							0.051
< $500	149	60.3	103	62.0	46	56.8	
$501 - $1,000	43	17.4	37	22.3	6	7.4	
$1,001 - $1,500	12	4.9	7	4.2	5	6.2	
Missing	43	17.4	19	11.4	24	29.6	
**On-campus meal plan**							0.902
Yes	56	22.7	40	24.1	16	19.8	
No	148	59.9	107	64.5	41	50.6	
Missing	43	17.4	19	11.4	24	29.6	
**Weight category by BMI**							0.416
Underweight	8	3.2	5	3.0	3	3.7	
Normal weight	112	45.3	78	47.0	34	42.0	
Overweight	41	16.6	33	19.9	8	9.9	
Obese	29	11.7	23	13.9	6	7.4	
Missing	57	23.1	27	16.3	30	37.0	
**Perceived health status**							0.438
Good/excellent	120	48.6	81	48.8	39	48.2	
Fair/poor	84	34.0	66	39.7	18	22.2	
Missing	43	17.4	19	11.4	24	29.6	
**Cook for self/others**							0.674
Often	108	43.7	75	45.2	33	40.7	
Sometimes	81	32.8	61	36.7	20	24.7	
Never	14	5.7	10	6.0	4	4.9	
Missing	44	17.8	20	12.0	24	29.6	
**Perceived cooking skills**							0.438
Good/excellent	136	55.0	95	57.2	41	50.6	
Fair/poor	68	27.6	52	31.3	16	19.8	
Missing	43	17.4	19	11.4	24	29.6	
**Marital status**							0.768
Not married	200	81.0	145	87.3	55	67.9	
Married	6	2.4	5	3.0	1	1.2	
Missing	41	16.6	16	9.6	25	30.9	
**Dependent children in household**							0.128
Yes	3	1.2	1	0.6	2	2.5	
No	205	83.0	150	90.4	55	67.9	
Missing	39	15.8	15	9.0	24	29.6	

Table 2. Perceived Barriers to On and Off-Campus Food Access Selected by Food Insecure Students With (n = 166) and Without (n=81) Disorders.*

On-Campus Students<br>Subscale	With Disorders<br>n	%	Without Disorders<br>n	%
**Knowledge**				
I don't know where to get information about food ingredients	5	3.0	3	3.7
I don't know how to ask for help	5	3.0	2	2.5
I don't know where to find places to get food	2	1.2	1	1.2
I don't have food preparation skills	1	0.6	1	1.2
Available food is not familiar to me	1	0.6	1	1.2
**Affective**				
I feel overwhelmed/stressed planning meals or making food choices	5	3.0	3	3.7
I feel embarrassed asking for help to access food	4	2.4	2	2.5
I am not interested/motivated to access food	3	1.8	1	1.2
**Food Access**				
Foods are not always healthy/nutritious	15	9.0	6	7.4
I can't find the foods I like	11	6.6	5	6.2
I have safety concerns	5	3.0	1	1.2
I need assistance accessing or preparing food	4	2.4	1	1.2
I require assistance with mobility that makes accessing food difficult	3	1.8	1	1.2
I don't have transportation	1	0.6	1	1.2
**Personal Concerns**				
Available foods do not taste good to me	14	8.4	5	6.2
Available foods do not support my special dietary needs	8	4.8	3	3.7
Available foods are not culturally appropriate	2	1.2	1	1.2
Available foods do not support my religious beliefs	1	0.6	1	1.2
My family doesn't want me to ask for help accessing food	1	0.6	0	0
**Practical Concerns**				
My meal plan runs out	17	10.2	10	12.3
I don't have time to eat regular meals	11	6.6	6	7.4
Food preparation is inconvenient	8	4.8	5	6.2
I don't have cooking equipment	8	4.8	4	4.9
I don't have enough money to purchase healthy/nutritious foods	7	4.2	3	3.7
I don't have time to purchase food	6	3.6	2	2.5

Off-Campus Students	With Disorders n	%	Without Disorders n	%
**Knowledge**				
I don't know where to get information about food ingredients	8	4.8	7	8.6
I don't know how to ask for help	7	4.2	2	2.5
I don't know where to find places to get food	3	1.8	2	2.5
I don't have food preparation skills	3	1.8	1	1.2
Available food is not familiar to me	3	1.8	1	1.2
**Affective**				
I feel overwhelmed/stressed planning meals or making food choices	21	12.7	6	7.4
I feel embarrassed asking for help to access food	4	2.4	2	2.5
I am not interested/motivated to access food	3	1.8	2	2.5
**Food Access**				
Foods are not always healthy/nutritious	9	5.4	3	3.7
I can't find the foods I like	6	3.6	2	2.5
I have safety concerns	5	3.0	2	2.5
I need assistance accessing or preparing food	5	3.0	2	2.5
I require assistance with mobility that makes accessing food difficult	3	1.8	1	1.2
I don't have transportation	2	1.2	1	1.2
**Personal Concerns**				
Available foods do not taste good to me	3	1.8	1	1.2
Available foods do not support my special dietary needs	2	1.2	1	1.2
Available foods are not culturally appropriate	2	1.2	1	1.2
Available foods do not support my religious beliefs	2	1.2	1	1.2
My family doesn't want me to ask for help accessing food	2	1.2	1	1.2
**Practical Concerns**				
My meal plan runs out	16	9.6	6	7.5
I don't have time to eat regular meals	11	6.6	5	6.2
Food preparation is inconvenient	9	5.4	2	2.5
I don't have cooking equipment	5	3.0	2	2.5
I don't have enough money to purchase healthy/nutritious foods	2	1.2	2	2.5
I don't have time to purchase food	0	0.0	1	1.2

* Some students selected more than one item.

Table 3. Coping Behaviors for Accessing Food Used by Food Insecure Students with (n=166) and without (n=81) Disorders.*

Strategy	With Disorders				Without Disorders			
	Sometimes/Often		Never/Seldom		Sometimes/Often		Never/Seldom	
	n	%	n	%	n	%	n	%
Brought food back to school	151	90.9	14	8.4	51	63	10	12.3
Ate less healthy foods to eat more	129	77.7	14	8.4	40	49.4	21	25.9
Ate smaller meals/portions	124	74.7	41	24.7	44	54.3	17	21.0
Planned menus	122	73.5	43	25.9	43	53.1	18	22.2
Stretched leftovers	109	65.6	56	33.7	38	46.9	23	28.4
Borrowed money from family or friends	105	63.2	60	36.2	29	35.8	32	39.5
Shared grocery and/or meal costs	102	61.4	64	38.6	31	38.2	30	37.0
Attended on-campus or community functions where there was free food	101	60.9	63	38.0	34	42	27	33.3
Ate more than normal when food was plentiful	97	58.4	65	39.1	33	40.7	28	34.6
Cut out food coupons	84	50.6	80	48.2	31	38.3	30	37.1
Held one or more jobs at the same time	79	47.6	86	51.8	24	29.6	37	45.7
Used a credit card to buy food	79	47.6	86	51.8	24	29.6	37	45.7
Accessed free food at food-related job	69	41.6	96	57.9	20	24.7	41	50.6
Took leftover food home from on-campus dining hall	47	28.3	117	70.4	14	17.2	47	58
Used less utilities	46	27.7	118	71.1	14	17.2	47	58
Sold personal possessions	44	26.5	121	72.9	8	9.8	53	65.4
Ate meals where you “pay what you can”	27	16.2	138	83.2	10	12.3	51	63.0
Attended free meal events in the community	25	15.0	141	85.0	12	14.8	49	60.5
Sold textbooks	23	13.8	140	84.3	3	3.7	58	71.6
Decreased medication dose or skipped medical appointments	16	9.6	149	89.7	2	2.5	59	72.8
Participated in a paid research study/clinical trial	15	12.0	150	90.4	3	3.7	58	71.6
Accessed food from a food pantry	14	8.4	151	90.9	8	9.9	53	65.4
Took fewer classes	8	4.8	156	94.0	1	1.2	60	74.1
Sold your blood/plasma	7	4.2	158	95.2	3	3.7	57	70.4
Dumpster Diving	6	3.6	158	95.2	5	6.2	56	69.1
Participated in federal or state food assistance program	5	3.0	160	96.4	3	3.7	58	71.6
Sold recreational or prescription drugs	5	3.0	160	96.4	2	2.5	59	72.9
Stole money	3	1.8	162	97.6	3	3.7	56	69.1

* Some students selected more than one strategy.
